# Analysis of risk factors for C5 nerve root paralysis after posterior cervical decompression

**DOI:** 10.1186/s12891-021-04434-y

**Published:** 2021-07-10

**Authors:** Bo Liu, Yanchen Chu, Jinfeng Ma, Xiaojie Tang, Junpeng Pan, Chunbing Wu, Xiao Chen, Chengliang Zhao, Zhijie Wang

**Affiliations:** 1 Department of Spinal Surgery, The Affiliated Hospital of Qingdao University, 266071 Qingdao, Shandong Province China; 2 Department of Spinal Surgery, Yantai Affiliated Hospital of Binzhou Medical University, 264100 Yantai, Shandong Province China

**Keywords:** C5 nerve root paralysis, PCSS, Risk factors, T2 hyperintensity, AUC

## Abstract

**Background:**

C5 nerve root paralysis is a nonnegligible complication after posterior cervical spine surgery (PCSS). The cause of its occurrence remains controversial. The purpose of this study was to analyse the incidence of and risk factors for C5 nerve root paralysis after posterior cervical decompression.

**Methods:**

We retrospectively analysed the clinical data of 640 patients who underwent PCSS in the Department of Orthopaedics, Affiliated Hospital of Qingdao University from September 2013 to September 2019. According to the status of C5 nerve root paralysis after surgery, all patients were divided into paralysis and normal groups. Univariate and multivariate analyses were used to determine the independent risk factors for C5 nerve root paralysis. A receiver operating characteristic (ROC) curve was used to demonstrate the discrimination of all independent risk factors.

**Results:**

Multivariate logistic regression analysis revealed that male sex, preoperative cervical spine curvature, posterior longitudinal ligament ossification, and preoperative C4/5 spinal cord hyperintensity were independent risk factors for paralysis, whereas the width of the intervertebral foramina was an independent protective factor for paralysis. The area under the curve (AUC) values of the T2 signal change at C4-C5, sex, cervical foramina width, curvature and posterior longitudinal ligament ossification were 0.706, 0.633, 0.617, 0.637, and 0.569, respectively.

**Conclusions:**

Male patients with C4-C5 intervertebral foramina stenosis, preoperative C4-C5 spinal cord T2 high signal, combined with OPLL, and higher preoperative cervical spine curvature are more likely to develop C5 nerve root paralysis after surgery. Among the above five risk factors, T2 hyperintensity change in C4-C5 exhibits the highest correlation with C5 paralysis and strong diagnostic power. It seems necessary to inform patients who have had cervical spine T2 hyperintensity before surgery of C5 nerve root paralysis after surgery, especially those with altered spinal cord T2 signals in the C4-C5 segment.

## Introduction

As the prevalence of long-term desk work increases, the prevalence of cervical spondylosis also increases yearly, which makes cervical spondylosis (CS) attract more attention. At present, the prevalence of CS in China is 3.8–17.6 % [1–3]. For patients with acquired spinal canal stenosis due to severe multisegment cervical myelopathy or ossification of the posterior longitudinal ligament (OPLL), posterior cervical spine surgery (PCSS) is a direct and effective treatment method, aiming to remove the lesion or expand the medullary cavity to directly relieve compression of the spinal cord so that the symptoms can be relieved and follow-up rehabilitation treatment can be achieved [4–6].

The most commonly used clinically methods for PCSS are single-door laminoplasty and total laminectomy decompression. However, postoperative complications, such as cervical 5 (C5) nerve root paralysis, epidural haematoma, axial pain, dural or nerve root injury, postoperative infection, and changes in cervical spine physiological flexion cannot be ignored [7]. Among them, C5 paralysis is of great significance for maintaining a good doctor-patient relationship and clinical effects. In clinical work, it has been noted that patients with T2 high signals on preoperative MRI are prone to C5 nerve root paralysis. Sex, intervertebral foramina stenosis, preoperative cervical spine curvature, and OPLL can also affect C5 nerve root paralysis. Based on the relevant literature, reports on the aetiology of C5 nerve root paralysis are still limited to the hypothesis. Different scholars have different opinions, and some even disagree [8, 9], which means that the exact aetiology of the complication remains unclear. Therefore, studies on the mechanism, influencing factors, prevention, and effective methods are of clinical significance to reduce the occurrence of C5 nerve root paralysis after PCSS.

## Materials and methods

### Inclusion and exclusion criteria

We retrospectively analysed the clinical data of 640 patients who underwent PCSS in the Department of Orthopaedics, Affiliated Hospital of Qingdao University from September 2013 to September 2019. Inclusion criteria: (1) patients with cervical spondylotic myelopathy who underwent cervical spine posterior single-door spinal canal expansion; (2) multisegmental spinal stenosis and spinal cord compression (≥ 3 segments); segmental kyphosis and instability.

Judgement of cervical 5 nerve root paralysis: (1) we performed electromyography on each patient before operation. patient has postoperative unilateral or bilateral biceps and deltoid muscle strength decline, and the decline is greater than 1 grade(MMT muscle strength standard); (2) with or without pain in the C5 nerve root innervation area and sensory function decline;

We can get the preoperative and postoperative cervical curvature of each patient by the patient’s X-ray examination.Measurement of cervical curvature: we make extension lines from the lower endplates of C2 and C7, and the angle of their perpendicular is the cervical curvature.The presence or absence of ossification of the posterior longitudinal ligament was clarified by CT scan examination, meanwhile, we could also measure the width of intervertebral foramen and laminectomy in each patient by CT scan examination.MRI can clarify the segment of spinal cord compression and the degree of compression, at the same time, we can also see whether the signal of spinal cord is changed.

## Surgical methods

All operations were performed by the same group of surgeons under general anaesthesia for tracheal intubation. Single-door laminoplasty takes the posterior midline incision and strips off the bilateral paravertebral muscles to expose the C3-7 bilateral lamina. According to the Magerl method, the needle insertion point is 1 mm inward from the midpoint of the lateral mass, sagittal to the head side 30°-50° angle, horizontal surface inclined 20°-25° angle outward, fixed with side block screws and pre-bent titanium rod, choose the side with heavier symptoms to open the door, and the side with the lighter symptoms as the door shaft side. The lamina was lifted to the side of the portal axis, and the adhesion structure between the ligamentum flavum and the dura mater was separated. The lamina was lifted to complete the decompression. Laminectomy and decompression surgery were the same as the nail placement method described above. The whole lamina is bitten from the inside of the articular joints. Rongeurs are used to expand the decompression range of the bilateral intervertebral foramen and nerve root canals, and bone fragments are implanted. Bone graft fusion is performed at small joints. After the operation, the incision was flushed. A drainage tube was placed for drainage, and the drainage tube was removed 24 to 48 h after the operation.

## Statistical methods

SPSS 21.0 statistical software was used to analyse and sort the data, and the count data were described by the number of cases and percentage (%). The statistical inferences between groups were tested by the Chi-squared test. Then, the measurement data were tested using the Shapiro-Wilk method for normality (*P* > 0.05). All data conformed to a normal distribution, so the measurement data are described by the mean ± standard deviation (M ± SD). Comparisons between the two groups were analysed using independent sample t-test.

Multivariate correlation analysis was analysed by a binary logistic regression model, we found that the VIF value of each variable was less than 5. There is no multicollinearity problem for the variables in the study. The results were expressed by the adjusted odds ratio (OR) and the corresponding 95 % confidence interval (CI). The area under the curve (AUC) was used to obtain the cut-off value of continuous variables, and the diagnostic performance of each index was obtained. The area under the curve (AUC) was used to evaluate the discrimination of indicator. The range is 0.5-1.0. AUC indicates the ability of diagnose diseases. The area under the ROC curve has been widely used in the discrimination evaluation of disease diagnosis model.The cut-off value means that the variable has the most appropriate sensitivity or specificity at this value, which can be used as a decision point for clinical diagnosis and analysis.When *P* < 0.05, the difference was statistically significant.

## Results

### General characteristics of the patient

A retrospective analysis of 640 patients with complete follow-up data who underwent posterior cervical surgery in the Orthopaedics Department of Qingdao University Affiliated Hospital from September 2013 to September 2019 was performed. There were 606 cases in the C5 nerve root non-paralysis group, including 338 males and 268 females, the mean age is 62.00 ± 10.29 years. The paralysis group included 34 cases with the mean age of 64.53 ± 9.23 years, including 28 males and 6 females. Among them, 459 cases were multisegment cervical spondylotic myelopathy, 48 cases were OPLL, 126 cases were cervical developmental spinal stenosis, and 7 cases were ankylosing spondylitis (Table 1).

**Table 1:** Comparison of general characteristics between the paralysis group and nonparalysis group

Variable	No C5 Palsy (*n *= 606)	C5 Palsy (*n* = 34)	χ^2^/t	P
Male/Female	338/268	28/6	χ^2^ = 9.288	**0.002**
Age(years)	62.00 ± 10.29	64.53 ± 9.23	t =-1.402	0.163
Diabetes	121(19.97)	7(20.59)	χ^2^ = 0.008	0.930
BMI (kg/m^2^)	25.57 ± 4.62	26.07 ± 4.41	t =-0.624	0.533
Foramen intervertebrale width	3.64 ± 1.05	3.22 ± 1.02	t = 2.307	**0.021**
OPLL	41(6.77)	7(20.59)	χ^2^ = 6.986	**0.008**
T2	192(31.68)	30(88.24)	χ^2^ = 45.446	**0.000**
T2 at C4-C5	107(17.66)	20(58.82)	χ^2^ = 34.301	**0.000**
Open angle (°)	58.88 ± 4.34	60.15 ± 4.19	t =-1.662	0.097
Laminectomy width	16.30 ± 2.57	15.59 ± 2.53	t = 1.579	0.115
Preoperative cervical curvature (°)	17.99 ± 4.50	20.24 ± 4.28	t =-2.841	**0.005**
Postoperative cervical curvature (°)	15.66 ± 3.52	16.53 ± 2.55	t =-1.897	0.065

*OPLL *posterior longitudinal ligament

## Univariate regression analysis results

Table 1 shows that the differences in the sex ratio, intervertebral foramina width, OPLL ratio, T2 and T2 at C4-C5 probability, and preoperative cervical curvature between the paralysis group and nonparalysis group were statistically significant (*P* < 0.05), and the male ratio, OPLL rate, T2 change rate, and preoperative cervical curvature in the paralysis group were significantly higher than those in the nonparalysis group. The width of the foramina in the paralysis group was significantly lower than that in the nonparalysis group. No significant differences in age, diabetes rate, BMI, open angle, laminectomy width, or postoperative cervical curvature were noted between the two groups (*P* > 0.05).

## Multivariate regression analysis results

Based on univariate analysis results, five indicators were used as independent variables, including sex ratio, intervertebral foramen width, OPLL rate, T2 at C4-C5, and preoperative cervical curvature. Multivariate regression analysis was performed using the occurrence of paralysis as the dependent variable (Table 2).

**Table 2:** Logistic regression analysis of paralysis and nonparalysis

Variables	β	OR	95 % CI	P
Gender	1.354	3.871	1.526–9.821	0.004
Foramen intervertebrale width	-0.402	0.669	0.462–0.969	0.034
OPLL	1.076	2.933	1.054–8.160	0.039
Preoperative cervical curvature	0.109	1.116	1.026–1.213	0.011
T2 at C4-C5	1.886	6.594	3.116–13.953	0.000

*β *coefficient estimation, *OR *odds ratio, which means the number of units increased by experimental variables, *CI *confidence interval, *OPLL *posterior longitudinal ligament

**Fig. 1:**
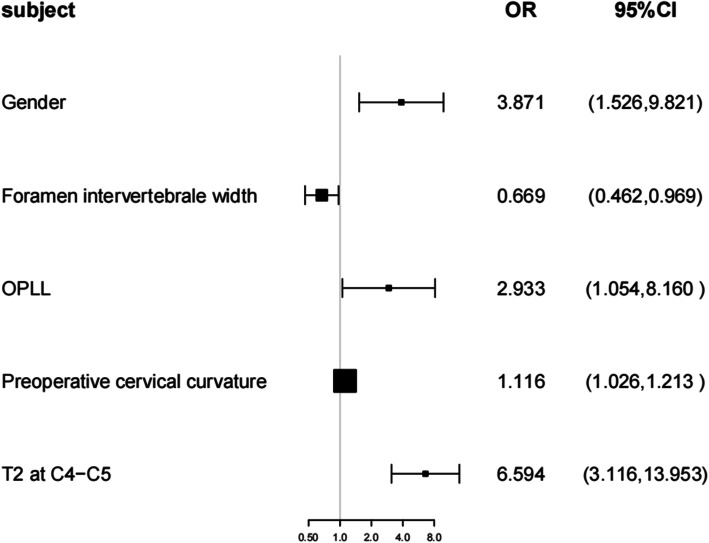
The results of multivariate regression analysis

Logistic regression analysis revealed a significant correlation between sex, intervertebral foramina width, preoperative cervical curvature, posterior longitudinal ligament ossification, T2 at C4-C5 signal changes, and paralysis (*P*<0.05). In addition, as shown in Fig. 1, sex, cervical curvature, posterior longitudinal ligament ossification, and T2 at C4-C5 signal changes were independent risk factors affecting paralysis (OR > 1), and intervertebral foramen width was an independent protective factor affecting paralysis (OR < 1).We drawn Forest plot(Fig. 1). In this way, the result is visualized graphically.

Patients with T2 signal changes at C4-C5 were 6.594-fold more likely to be paralyzed (OR = 6.594) than those without changes. Men were 3.871-fold more likely to be paralyzed than women (OR = 3.871). The probability of paralysis in patients with OPLL was 2.933 times that of patients without OPLL (OR = 2.933). For every unit increase in the width of the intervertebral foramen, the probability of paralysis in patients was reduced by 0.331 times (OR = 0.669). For every unit increase in the degree of preoperative cervical curvature, the probability of paralysis increased by 0.116 times (OR = 1.116). The multivariate regression conclusion supports the results of univariate analysis.

### ROC curve

**Fig. 2:**
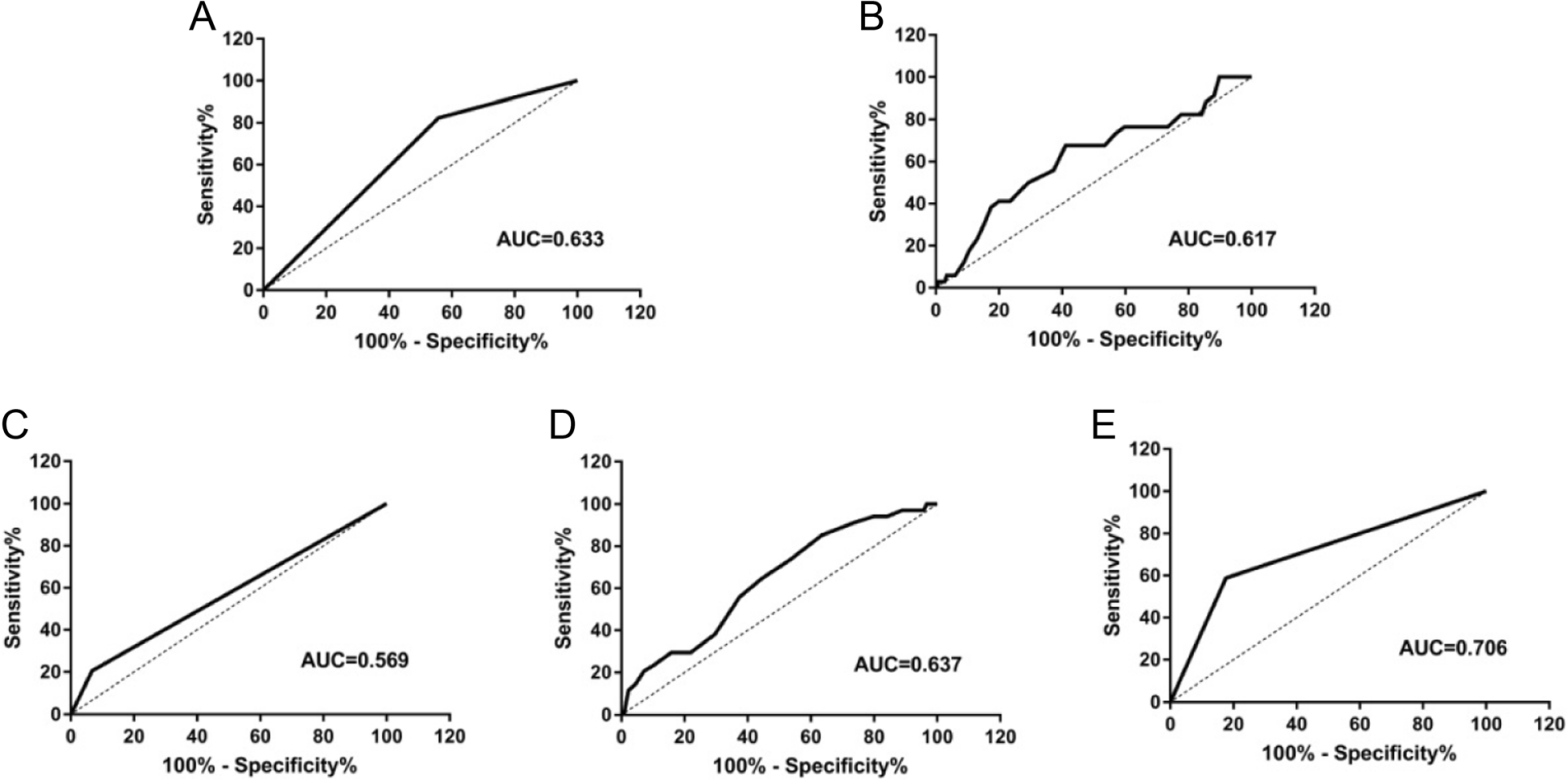
The AUC of the five independent factors. (A) Sex; (B) foramen intervertebral width; (C) OPLL; (D) preoperative cervical curvature; (E) T2 at C4-C5

To further obtain the value of sex, intervertebral foramen width, preoperative cervical curvature, OPLL, and T2 signal change for the diagnosis of paralysis, we chose ROC curve analysis (Table 3),We also plot the ROC curve for each meaningful variable (Fig. 2).

**Table 3:** ROC results of multiple regression analysis factors

Variables	AUC	95 % CI	Cut-off	Sensitivity	Specificity
Gender	0.633	0.547–0.719			
Foramen intervertebral width	0.617	0.516–0.718	3.350	0.676	0.589
OPLL	0.569	0.462–0.677			
Preoperative cervical curvature	0.637	0.547–0.726	16.50	0.853	0.365
T2 at C4-C5	0.706	0.606–0.806			

*β *coefficient estimation, *OR *odds ratio, which means the number of units increased by experimental variables, *CI *confidence interval, *OPLL *posterior longitudinal ligament

The AUC of the T2 signal change index at C4-C5 was 0.706, These results indicate that the T2 signal change index at C4-C5 exhibits a certain diagnostic efficacy. The AUC of the sex index was 0.633. It indicated that the sex index was generally effective in diagnosing diseases. Cervical foramina width (AUC = 0.617), curvature (AUC = 0.637), and posterior longitudinal ligament ossification (AUC = 0.569) were not effective in diagnosing diseases (Fig. 2).Meanwhile, the diagnostic effect of the width of the intervertebral foramen was average, and the corresponding cut-off value was 3.35 mm.This shows that “the width of intervertebral foramen” has the best specificity and specificity at this value.For the preoperative cervical curvature, “16.50 mm” is the best value.

## Discussion

C5 nerve root paralysis is one of the common complications after PCSS, especially after cervical spine single-door laminoplasty and laminectomy. Sakaura et al. [10] reviewed the literature from 1986 to 2002 and found that most of the complications of this operation occurred within 1 day to 1 week after surgery and a few occurred within 2 to 4 weeks after surgery. At present, most researchers define it as a new deltoid muscle and/or biceps brachii paralysis without the aggravation of spinal cord symptoms after cervical decompression with a decrease in muscle strength of greater than grade 1 [11]. According to the literature reports, the average incidence of C5 nerve root paralysis is 4.6 % after removal of cervical spinal cord compression, 4.3 % after anterior decompression and fusion, and 5.8 % (1.4∼18.4 %) after posterior decompression [12]. Although its incidence is high, the prognosis is generally good. It has been reported in the literature that greater than 70 % of patients can fully recover within 4 to 5 months after surgery, and most of them recover naturally [13–15].

Patients with a high T2 signal at C4-C5 before surgery were 6.594 times more likely to develop paralysis than patients without a change (OR = 6.594). Compared with several other factors, this parameter exhibits strong diagnostic efficacy. In other words, if the patient has MRI-T2WI intramedullary hyperintensity at the C4-C5 segment before surgery, the patient is more likely to have C5 nerve root paralysis after surgery.

This finding may be due to cerebrospinal fluid reflux disorder and/or spinal cord blood flow disorder after spinal cord compression, resulting in spinal cord oedema and ischaemia. With a high T2WI signal on MRI, local compression is suddenly relieved during spinal cord surgery, causing ischaemia-reperfusion injury [16–18]. Many studies [13, 14] have confirmed that patients with intramedullary hyperintensity on T2WI typically have a worse prognosis than patients without intramedullary hyperintensity even after surgery. Hasegawa K et al. [19] noted that grey matter degeneration (T2 high signal) can lead to C5 nerve root paralysis after cervical spine surgery, which is caused by local spinal cord reperfusion injury. This finding is consistent with the results of our study. Similarly, Seichi et al. [20] further conducted a statistical study, indicating that postoperative MRI-T2 weighted image changes are statistically significantly correlated with upper limb paralysis symptoms.

Men were 3.871 times more likely to have paralysis than women (OR = 3.871). This finding suggests that men are more likely to suffer from this complication after cervical surgery. There was no significant difference in the comparison of the cervical foramen area between men and women.The study showed that men had a higher risk of developing postoperative C5 palsy than women,This may be due to other factors than women having a narrower intervertebral foramen[21].The OPLL of the paralysis group was 20.59 %, and the OPLL of the nonparalysis group was 6.77 %. A significant difference was noted in univariate analysis (*P* = 0.008). However, Sakaura [10] found that the probability of C5 nerve root paralysis due to OPLL is 2.1 to 14 %. Some scholars believe that OPLL itself is not a risk factor for C5 paralysis. We believe that the existence of OPLL possibly causes stenosis of the intervertebral foramina, which can lead to nerve root paralysis after surgery.

For every unit increase in cervical spine curvature before surgery, the patient’s probability of paralysis increased by 0.116 times (OR = 1.116). This findings may be attributed to the fact that the facet joints at C4-C5 are more forward than other segments. In patients with large cervical curvature, the C5 segment is typically located at the apex of the decompression zone during open cervical surgery, and the degree of spinal cord retraction is the largest in the C5 segment [22]. Additionally, the deltoid muscle is only dominated by the C5 segment [23]; thus, the possibility of nerve root paralysis after surgery will increase.

Compared with the width of the C4-C5 intervertebral foramen in the nonparalysis group (3.64 ± 1.05 mm), the width of the C4-C5 intervertebral foramen in the paralysis group was narrower (3.22 ± 1.02 mm). Comparative analysis of the two was statistically significant (*P* = 0.021). S. Imagama et al. [24] also found that C4/5 intervertebral foramina stenosis, C5 superior articular process hypertrophy, and C4/5 spinal cord rotation patients exhibit an increased probability of C5 nerve root paralysis. Anatomically, the length of the ventral branch of C5 is shorter than that of other segments [25], and it is easily damaged during the stretching process. Careful preoperative evaluation of this risk factor and proper foraminal incision during the operation may reduce postoperative C5 paralysis.

Current measures to prevent nerve root paralysis include intraoperative neuroelectrophysiological testing and preventive foraminotomy. Bradford L. Currier et al. [14] believe that the use of nerve and spinal cord monitoring during cervical decompression can effectively prevent nerve root damage. However, intraoperative monitoring is not routinely used in our patients, and the penetration rate is too low. Another method used to avoid C5 nerve paralysis is to perform preventive foraminal incision during laminoplasty or laminectomy. When Katsumi et al. [8] performed preventive C4/5 intervertebral foramina decompression during posterior laminoplasty in 141 patients, only 2 patients developed symptoms of C5 nerve root paralysis after the operation, accounting for approximately 1.4 %, which is far less than the incidence of C5 nerve root paralysis in previous studies.

However, this study also has limitations. First, the current study is a small-scale retrospective data analysis of a single institution using a small sample size and lacking multicentre collaborative research with other institutions. Second, although we confirmed that preoperative T2 hyperintensity of the spinal cord is a risk factor for posterior cervical surgery, we did not use a quantitative method to measure the change in spinal cord signal. We only rely on subjective identification of whether a high T2 signal appears in the preoperative MRI report; thus, this process is subject to deviation and subjectivity.

## Conclusions

Male patients with C4-C5 intervertebral foramina stenosis, preoperative C4-C5 spinal cord T2 high signal, combined with OPLL, and higher preoperative cervical spine curvature are more likely to develop C5 nerve root paralysis after surgery. Among the above five risk factors, T2 hyperintensity change in C4-C5 exhibits the highest correlation with C5 paralysis and strong diagnostic power. For those patients who have had cervical spine T2 hyperintensity before surgery, C5 nerve root paralysis should be assessed after surgery, especially for those with altered spinal cord T2 signals in the C4-C5 segment.

## Data Availability

The data used and analyzed during the current study are available in anonymized form from the corresponding author on reasonable request.

## Abbreviations

PCSS: Posterior cervical spine surgery
ROC: Receiver operating characteristic
CS: Cervical spondylosis
OPLL: Posterior longitudinal ligament
C5: Cervical 5
OR: Odds ratio
AUC: Area under the curve
CI: Confidence interval
VIF: Variance inflation factors
